# A PET-Derived SUVmax-to-Albumin Ratio Predicts Recurrence After Neoadjuvant FLOT in Gastric Cancer

**DOI:** 10.3390/medicina62050964

**Published:** 2026-05-14

**Authors:** Emine B. Eniseler, Bartu Çetin, Ferhat Ekinci, Mustafa Sahbazlar, Atike P. Erdogan

**Affiliations:** 1 Department of Medical Oncology, Faculty of Medicine, Celal Bayar University, 45140 Manisa, Türkiye; drbihtereniseler@gmail.com (E.B.E.); drferhatekinci@hotmail.com (F.E.); m_sahbazlar@hotmail.com (M.S.); 2 Department of General Surgery, Manisa City Hospital, 45040 Manisa, Türkiye; drbartucetin@gmail.com

**Keywords:** FDG PET/CT, gastric cancer, metabolic–nutritional index

## Abstract

*Background and Objectives*: Although perioperative FLOT improves outcomes in locally advanced gastric cancer, postoperative recurrence remains frequent. This study evaluated the prognostic value of a PET-derived SUVmax/albumin ratio integrating tumor metabolism and nutritional status in patients treated with neoadjuvant FLOT. *Materials and Methods*: This retrospective single-center study included patients with gastric adenocarcinoma treated with neoadjuvant FLOT followed by curative gastrectomy between January 2017 and May 2025, using data obtained from existing medical records after ethical approval. Pre-treatment SUVmax from ^18^F-FDG PET/CT and serum albumin were recorded to calculate the SUVmax/albumin ratio. Progression-free survival (PFS) was the primary endpoint. Prognostic factors were analyzed using Cox regression, and discriminatory performance was assessed using receiver operating characteristic (ROC) analysis. *Results*: A total of 104 patients were included. During a median follow-up of 28 months (IQR, 16–46 months), 46 patients (44.2%) developed recurrence. Patients with recurrence had significantly lower serum albumin levels and higher PET SUVmax values and PET SUVmax/albumin ratios (all *p* < 0.001). In multivariable analysis, the PET SUVmax/albumin ratio remained significantly associated with PFS (HR 1.71; 95% CI 1.33–2.21; *p* < 0.001). ROC analysis demonstrated moderate discriminatory performance (AUC 0.733; 95% CI 0.638–0.815), while additional time-dependent ROC analyses yielded AUC values of 0.732 (95% CI 0.611–0.836) at 1 year and 0.829 (95% CI 0.694–0.947) at 3 years. Exploratory comparative analyses demonstrated that both PET SUVmax and serum albumin retained statistical significance when evaluated simultaneously in the same multivariable model. *Conclusions*: The pre-treatment PET SUVmax/albumin ratio was significantly associated with PFS in patients with gastric cancer treated with neoadjuvant FLOT and may represent an exploratory composite prognostic biomarker requiring further prospective validation.

## 1. Introduction

Gastric cancer remains a major global health burden and continues to rank among the leading causes of cancer-related mortality. Although incidence and mortality have declined in certain regions, overall outcomes remain unsatisfactory, particularly in patients presenting with locally advanced disease [1]. Even after curative-intent gastrectomy, postoperative recurrence is frequently observed, underscoring the ongoing need for improved prognostic stratification in surgically treated patients. Recent systematic reviews and meta-analyses have highlighted that disease recurrence continues to represent a significant clinical challenge despite advances in perioperative management [2].

Robust evidence from randomized controlled trials has established perioperative chemotherapy as the standard of care for patients with locally advanced gastric and gastroesophageal junction adenocarcinoma. In the pivotal FLOT4 trial, the perioperative FLOT regimen—consisting of fluorouracil, leucovorin, oxaliplatin, and docetaxel—demonstrated superior overall and disease-free survival compared with previous standard regimens [3]. Accordingly, current international guidelines recommend perioperative FLOT chemotherapy as the preferred treatment approach in eligible patients [4,5]. Despite this strategy, a substantial proportion of patients treated with neoadjuvant FLOT followed by curative gastrectomy still develop disease recurrence, reflecting significant biological heterogeneity and variability in treatment response.

From a surgical perspective, improved preoperative risk stratification may contribute to a better understanding of recurrence patterns following curative-intent treatment. Several conventional clinicopathological features—such as pathological lymph node involvement (ypN), lymphovascular invasion (LVI), perineural invasion (PNI), tumor localization, and histological subtype—have been associated with recurrence risk [5]. However, systematic reviews indicate that these parameters provide limited predictive accuracy in the neoadjuvant setting, and importantly, become available only after surgical resection [6]. This limitation has prompted growing interest in pre-treatment biomarkers capable of integrating tumor biology with host-related factors.

Metabolic imaging with ^18^F-fluorodeoxyglucose positron emission tomography/computed tomography (^18^F-FDG PET/CT) provides functional insights into tumor aggressiveness. Previous meta-analyses have demonstrated that higher maximum standardized uptake values (SUVmax) are significantly associated with adverse survival outcomes and increased recurrence risk in gastric cancer [7,8]. However, SUVmax alone does not reflect patient-related nutritional or systemic inflammatory status, both of which influence disease progression and treatment tolerance.

Nutritional status markers such as serum albumin and body mass index (BMI) are well-recognized independent prognostic indicators in gastric cancer, as confirmed by systematic reviews and pooled analyses [9,10]. Additionally, composite indices integrating nutritional or inflammatory markers have shown prognostic utility across multiple solid tumors. Despite these observations, data on combined metabolic–nutritional indices incorporating PET-derived parameters remain scarce.

To date, the prognostic relevance of integrating PET SUVmax with serum albumin levels for predicting postoperative recurrence in gastric cancer patients treated with neoadjuvant FLOT chemotherapy has not been clearly defined. Therefore, this study aimed to evaluate whether the pre-treatment PET SUVmax/albumin ratio could predict postoperative recurrence and progression-free survival (PFS) in patients with locally advanced gastric cancer undergoing neoadjuvant FLOT chemotherapy followed by curative gastrectomy.

## 2. Materials and Methods

This retrospective single-center cohort study was conducted in accordance with the Strengthening the Reporting of Observational Studies in Epidemiology (STROBE) guidelines. Trial registration was not applicable due to the observational study design. All data were retrospectively obtained from existing medical records after ethical approval.

Patients with histopathologically confirmed gastric adenocarcinoma who received neoadjuvant FLOT chemotherapy followed by curative-intent gastrectomy between January 2017 and May 2025 at Manisa Celal Bayar University Hospital were eligible for inclusion.

Patients were excluded if they underwent upfront surgery, received neoadjuvant regimens other than FLOT, had metastatic or unresectable disease, or had incomplete clinical, pathological, or follow-up data.

### 2.1. Data Collection and Clinical Assessment

Demographic characteristics, anthropometric measurements, and comorbidities were retrospectively obtained from patient files and electronic medical records. Performance status at diagnosis was assessed using the Eastern Cooperative Oncology Group performance status (ECOG-PS) scale and categorized from 0 (fully active) to 4 (completely disabled) [11].

### 2.2. Clinical Staging

Clinical staging was performed using contrast-enhanced thoracoabdominal computed tomography (CT), upper gastrointestinal endoscopy, and 18F-FDG PET/CT imaging. Lymph node involvement was assessed based on radiologic criteria, including lymph node enlargement, abnormal morphology (e.g., rounded contour or loss of fatty hilum), or increased FDG uptake suggestive of metastatic involvement. Distant metastases were evaluated using contrast-enhanced CT and PET/CT imaging. Patients with radiological or metabolic evidence of metastatic disease were excluded from curative-intent surgery. All staging assessments were reviewed in a multidisciplinary tumor board setting according to institutional practice.

### 2.3. Pathological Evaluation

Pathological data included tumor differentiation grade, presence of LVI and PNI, type of surgical resection (total or subtotal gastrectomy), number of resected lymph nodes, and number of pathologically positive lymph nodes. Post-neoadjuvant pathological staging was performed using the ypT and ypN categories. All pathological staging procedures were conducted in accordance with the American Joint Committee on Cancer (AJCC) Tumor–Node–Metastasis (TNM) staging system, 8th edition, appropriate to the time of diagnosis and treatment [12].

### 2.4. PET/CT Acquisition, Image Analysis, and Biomarker Assessment

All patients underwent pre-treatment ^18^F-FDG PET/CT imaging at a single institution using standardized acquisition protocols. Patients fasted for at least 6 h prior to imaging, and blood glucose levels were measured before ^18^F-FDG administration. PET/CT examinations were performed when blood glucose levels were below 200 mg/dL, in accordance with the established international PET imaging guidelines. Image acquisition was performed approximately 60 min after tracer injection according to institutional clinical practice standards. Although lower glucose levels are preferable for optimal image quality, the <200 mg/dL threshold is widely accepted in routine clinical practice.

SUVmax of the primary tumor was measured on attenuation-corrected images using region-of-interest (ROI) analysis. Image interpretation and quantitative measurements were performed by experienced nuclear medicine physicians who were blinded to clinical outcomes in order to minimize bias.

SUVmax was selected as the primary metabolic parameter due to its widespread clinical availability, reproducibility, and ease of integration into routine nuclear medicine workflows. To combine tumor metabolic activity with host nutritional status, the PET SUVmax/albumin ratio was calculated using serum albumin levels obtained at the time of diagnosis.

HER2 status was evaluated by immunohistochemistry, and fluorescence in situ hybridization was performed in cases with an equivocal immunohistochemical score (2+). Microsatellite instability (MSI) status was determined by immunohistochemical assessment of MLH1, MSH2, MSH6, and PMS2 protein expression.

### 2.5. Follow-Up and Outcomes

Patients were followed at regular intervals according to institutional clinical practice. Follow-up evaluations were performed every 3 months during the first 2 years after surgery and every 6 months thereafter. Each follow-up visit included physical examination, laboratory tests, and contrast-enhanced CT imaging.

Recurrence was defined as the appearance of new locoregional or distant lesions detected on contrast-enhanced CT imaging that were consistent with malignant disease. Radiological recurrence was diagnosed based on the development of new lesions or progressive enlargement of previously detected suspicious lesions demonstrating imaging characteristics suggestive of malignancy. When clinically feasible, suspected recurrent lesions were confirmed by histopathological examination. In cases where biopsy was not feasible, recurrence was determined based on consistent radiological progression findings and clinical correlation.

PFS was calculated from the date of surgery to the date of first documented recurrence or death from any cause, whichever occurred first. Patients without recurrence were censored at the date of last follow-up. Overall survival (OS) was calculated from the date of surgery to death from any cause or last follow-up.

### 2.6. Statistical Analysis

Statistical analyses were performed using IBM SPSS Statistics version 24.0. Continuous variables were assessed for normality using visual and analytical methods and were expressed as the mean ± standard deviation or median (interquartile range [IQR]), as appropriate. Categorical variables were summarized as frequencies and percentages. Comparisons between groups were performed using the Student’s *t*-test or Mann–Whitney U test for continuous variables and the chi-square test or Fisher’s exact test for categorical variables, as appropriate.

PFS was defined as the time from surgery to documented recurrence, progression, or death from any cause. OS was defined as the time from surgery to death from any cause or last follow-up. Survival curves were estimated using the Kaplan–Meier method and compared using the log-rank test. Prognostic factors associated with PFS and exploratory OS analyses were evaluated using univariate and multivariate Cox proportional hazards regression models. Given the limited number of progression events, parsimonious multivariable models were constructed to reduce the risk of overfitting. Variables included in multivariable analyses were selected based on clinical relevance and univariate significance. Proportional hazards assumptions were assessed graphically. Before multivariable analysis, collinearity among covariates was evaluated using variance inflation factors, and no significant multicollinearity was detected.

Receiver operating characteristic (ROC) curve analysis was performed to evaluate the discriminatory performance of the PET SUVmax/albumin ratio and to identify an exploratory cutoff value using the Youden index. Additional exploratory time-dependent ROC analyses and Harrell’s C-index calculations were also performed to assess prognostic discrimination at different follow-up time points. Statistical significance was defined as a two-sided *p*-value < 0.05.

### 2.7. Ethical Approval

The study was approved by the Manisa Celal Bayar University Clinical Research Ethics Committee (Approval No. 20.478.486; 4 June 2025). Informed consent was waived due to the retrospective design, and the study was conducted in accordance with the Declaration of Helsinki.

## 3. Results

### 3.1. Patient Characteristics and Recurrence

A total of 104 patients who underwent curative surgery after neoadjuvant FLOT chemotherapy were included. During a median follow-up of 28 months (IQR, 16–46 months), disease recurrence occurred in 46 patients (44.2%). Patient characteristics according to recurrence status are summarized in Table 1.

Age, sex distribution, and ECOG-PS were comparable between patients with and without recurrence (all *p* > 0.05). In contrast, BMI values were significantly lower in patients who developed recurrence, and the proportion of patients with BMI ≥ 25 was higher in the non-recurrence group (*p* = 0.015). HER2 and microsatellite instability status did not differ between groups.

Tumor localization differed significantly according to recurrence status, with diffuse-type tumors more frequently observed in the recurrence group and cardia localization more common in patients without recurrence (*p* = 0.007). Histological subtype distribution was similar between groups, whereas ypT stage differed significantly according to recurrence status (*p* = 0.014). Tumor differentiation showed a trend toward significance (*p* = 0.057).

Regarding pathological features, LVI and PNI were significantly more frequent in patients with recurrence (*p* = 0.001 and *p* = 0.014, respectively). Post-neoadjuvant ypN stage was strongly associated with recurrence, with ypN3 disease more prevalent in the recurrence group and ypN0 status more common in patients without recurrence (*p* < 0.001). Total gastrectomy was more commonly performed in the recurrence group, whereas subtotal gastrectomy was more frequent in patients without recurrence (*p* = 0.014). Most patients underwent D2 lymphadenectomy (98.1%). Patients with recurrence also had significantly higher numbers of pathologically positive lymph nodes and higher total retrieved lymph node counts (*p* < 0.001 and *p* = 0.018, respectively).

Patients who developed recurrence had significantly higher PET SUVmax values and PET SUVmax/albumin ratios, along with lower serum albumin levels (all *p* < 0.001). Baseline renal and hepatic function parameters, including creatinine, GFR, ALT, ALP, bilirubin, and LDH levels, did not significantly differ according to recurrence status (all *p* > 0.05). Similarly, baseline CRP levels were comparable between patients with and without recurrence. Baseline comorbidity profiles, including chronic obstructive pulmonary disease (COPD) (*p* = 0.797), diabetes mellitus (*p* = 0.220), and coronary artery disease (CAD) (*p* = 0.460), also did not significantly differ according to recurrence status.

In addition, the recurrence group received fewer adjuvant FLOT cycles compared with the non-recurrence group (*p* = 0.001). FLOT-related grade 1–2 toxicities were observed in 47 patients (45.2%), whereas grade 3–4 toxicities occurred in 10 patients (9.6%). The distribution of treatment-related toxicities did not significantly differ between patients with and without recurrence (grade 1–2: *p* = 0.478; grade 3–4: *p* = 0.506).

### 3.2. Survival and Cox Regression Analyses

During follow-up, 46 progression events occurred, with a median PFS of 36 months. One-, three-, and five-year PFS rates were 70.9%, 49.7%, and 45.9%, respectively. In univariate Cox analysis, BMI < 25, LVI positivity, PNI positivity, tumor localization, type of surgical resection, higher PET SUVmax, and higher PET SUVmax/albumin ratio were associated with worse PFS.

The results of the univariate and multivariate Cox regression analyses are summarized in Table 2. Given the limited number of progression events, a parsimonious multivariable Cox model was constructed to reduce the risk of overfitting. In multivariate analysis, tumor localization, type of surgical resection, and the PET SUVmax/albumin ratio remained significantly associated with PFS. The PET SUVmax/albumin ratio remained associated with an increased risk of progression or recurrence after adjustment for other covariates (HR 1.71; 95% CI 1.33–2.21; *p* < 0.001).

Based on ROC curve analysis, the PET SUVmax/albumin ratio demonstrated moderate discriminatory ability for PFS events (AUC 0.733; 95% CI 0.638–0.815). The optimal cutoff value determined by the Youden index was 2.9, yielding a modest sensitivity (50.0%) but high specificity (91.4%). Time-dependent ROC analyses demonstrated AUC values of 0.732 (95% CI 0.611–0.836) at 1 year and 0.829 (95% CI 0.694–0.947) at 3 years for the PET SUVmax/albumin ratio. Harrell’s C-index also demonstrated moderate prognostic discrimination for PFS (C-index 0.698; 95% CI 0.608–0.785).

**Figure 1:**
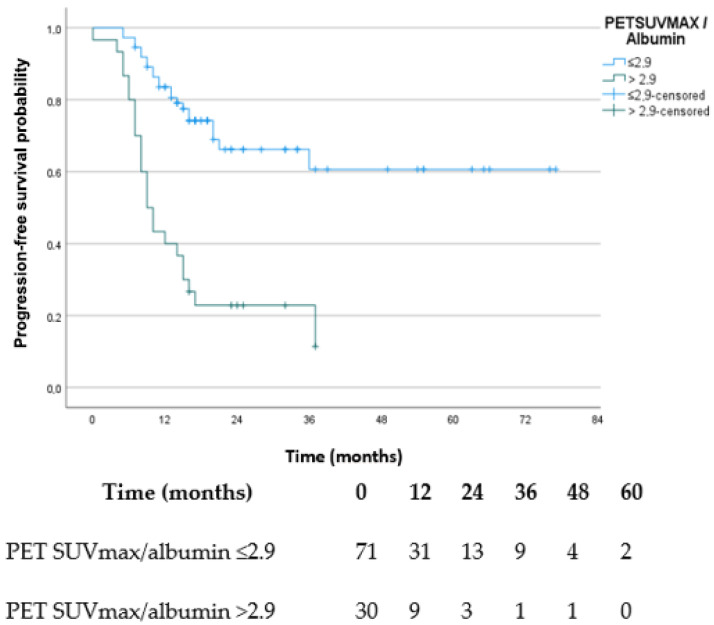
Kaplan–Meier curves for progression-free survival according to the PET SUVmax/albumin ratio cutoff value (≤2.9 vs. >2.9). Patients with a PET SUVmax/albumin ratio > 2.9 demonstrated significantly shorter progression-free survival compared with those with a ratio ≤ 2.9 (log-rank *p* < 0.001). Numbers at risk are shown below the figure.

Kaplan–Meier analysis demonstrated significantly shorter PFS in patients with a PET SUVmax/albumin ratio > 2.9 compared with those with a ratio ≤ 2.9 (Figure 1).

Additional exploratory comparative multivariable Cox regression analyses were performed using the same parsimonious adjustment strategy applied in the primary multivariable model (Table 3). Model 1 evaluated PET SUVmax, Model 2 evaluated serum albumin, Model 3 simultaneously included both PET SUVmax and serum albumin in the same multivariable model, and Model 4 evaluated the PET SUVmax/albumin ratio. When entered simultaneously into the same multivariable model, both PET SUVmax and serum albumin retained statistical significance.

### 3.3. Exploratory Overall Survival Analysis

An exploratory OS analysis was also performed. During follow-up, 41 deaths (39.4%) occurred. Patients who developed recurrence demonstrated significantly higher mortality rates compared with those without recurrence (82.6% vs. 3.4%, *p* < 0.001). A higher PET SUVmax/albumin ratio was significantly associated with worse OS in univariate Cox analysis (HR 1.44; 95% CI 1.22–1.71; *p* < 0.001). When categorized according to the ROC-derived cutoff, patients with a PET SUVmax/albumin ratio > 2.9 had significantly shorter OS compared with those with a ratio ≤ 2.9 (median OS, 15.0 months vs. not reached; log-rank *p* < 0.001). In a parsimonious multivariable Cox model adjusted for surgical resection type, LVI, and ypN3 status, the PET SUVmax/albumin ratio remained significantly associated with worse OS (HR 1.44; 95% CI 1.19–1.76; *p* < 0.001).

## 4. Discussion

In this retrospective single-center study, we evaluated the clinical, pathological, and imaging-derived variables associated with recurrence in patients with gastric cancer treated with neoadjuvant FLOT chemotherapy. A principal finding of this study was that the pre-treatment PET SUVmax/albumin ratio remained significantly associated with PFS after adjustment for selected clinicopathological covariates.

Despite advances in multimodal treatment strategies, gastric cancer remains a major contributor to cancer-related mortality worldwide [1]. The FLOT regimen has become the standard perioperative approach for resectable, locally advanced gastric and gastroesophageal junction cancers, based on demonstrated survival benefits [2,3,4]. However, postoperative recurrence remains common even in patients receiving optimal systemic therapy, underscoring the need for robust pre-treatment biomarkers capable of investigating risk stratification.

Consistent with previous reports, pathological variables such as LVI, PNI, and post-neoadjuvant nodal status were associated with recurrence in univariate analyses [3,4]. Nevertheless, several of these parameters lost statistical significance in multivariate modeling. This observation highlights a critical limitation of postoperative pathological markers: they become available only after surgical resection and may therefore have limited utility for pre-treatment risk assessment in patients undergoing neoadjuvant therapy.

A central contribution of our study is the integration of tumor metabolic activity with host-related nutritional status. FDG uptake assessed by PET/CT reflects tumor glucose metabolism, cellular proliferation, and hypoxic adaptation, and elevated SUVmax values have consistently been linked to unfavorable outcomes in gastric cancer [5,6]. However, FDG uptake in gastric cancer is biologically heterogeneous and may vary according to histological subtype, tumor cellularity, mucin content, and glucose metabolic phenotype. In particular, signet-ring cell and mucinous tumors have been reported to demonstrate relatively lower FDG avidity, potentially influencing SUV-based measurements and prognostic interpretation. Although histological subtype was evaluated in our cohort, the retrospective design and limited subgroup sizes precluded more detailed stratified analyses according to PET metabolic phenotypes. Therefore, the prognostic performance of the PET SUVmax/albumin ratio may differ across biological subtypes of gastric cancer and warrants further investigation in larger prospective cohorts. SUVmax alone captures only tumor-intrinsic metabolic activity and does not account for host-related biological vulnerability, which may influence recurrence patterns and therapeutic resilience.

Serum albumin, conversely, is a well-established surrogate marker of nutritional status, systemic inflammation, and physiological reserve [7,8,9]. Hypoalbuminemia has been associated with inferior survival, increased perioperative complications, and reduced tolerance to systemic treatment in gastric malignancies [8,9]. However, serum albumin levels may also be influenced by non-oncologic factors such as nutritional status, systemic inflammation, hepatic dysfunction, renal disease, and chronic comorbid conditions, which should be considered when interpreting albumin-based indices. Nevertheless, baseline renal and hepatic function parameters, including creatinine, GFR, ALT, ALP, bilirubin, and LDH levels, did not significantly differ according to recurrence status in our cohort. This observation suggests that the prognostic association of the PET SUVmax/albumin ratio may not be solely attributable to overt hepatic or renal dysfunction. In addition, baseline CRP levels did not significantly differ between patients with and without recurrence, suggesting that the observed association was not solely driven by baseline systemic inflammatory status. Similarly, baseline comorbidity profiles, including COPD, diabetes mellitus, and CAD, were comparable between recurrence groups. At our institution, patients routinely receive dietitian support and periodic nutritional assessment as part of standard multidisciplinary oncologic care during systemic treatment and perioperative follow-up. Treatment-related toxicity profiles were comparable between recurrence groups, although patients with recurrence received fewer adjuvant FLOT cycles.

By combining SUVmax with serum albumin, the PET SUVmax/albumin ratio provides a composite metric incorporating both tumor-related metabolic information and host-related nutritional/inflammatory status. However, because comprehensive inflammatory, sarcopenic, and cachexia-related assessments were not uniformly available, the PET SUVmax/albumin ratio should be interpreted primarily as a composite prognostic marker reflecting both tumor-related metabolic activity and host-related nutritional/inflammatory status rather than as a direct causal biomarker of tumor biology alone. In our multivariate analysis, the PET SUVmax/albumin ratio remained significantly associated with PFS, whereas several conventional pathological variables lost statistical significance after adjustment.

These findings align with emerging evidence suggesting that metabolic–nutritional or metabolic–inflammatory composite indices may outperform single-parameter models in predicting recurrence and survival across various solid tumors [13,14,15,16,17]. Importantly, our results extend this concept to patients treated with neoadjuvant FLOT, a clinically relevant subgroup in which prognostic biomarkers remain incompletely defined.

Additional exploratory comparative multivariable analyses demonstrated that both PET SUVmax and serum albumin retained statistical significance when entered simultaneously into the same multivariable model. These findings support the potential prognostic relevance of both metabolic and host-related nutritional parameters in patients treated with neoadjuvant FLOT chemotherapy.

ROC analysis identified an optimal PET SUVmax/albumin ratio cutoff value of 2.9, characterized by high specificity (91.4%) but modest sensitivity (50.0%). In addition to conventional ROC analysis, both time-dependent ROC analyses and Harrell’s C-index demonstrated moderate discriminatory performance for PFS [18]. These findings suggest that the PET SUVmax/albumin ratio may have potential prognostic relevance in patients treated with neoadjuvant FLOT chemotherapy; however, further prospective validation is required before any clinical application.

Several limitations merit consideration. The retrospective single-center design introduces potential selection bias and limits generalizability. Although PET/CT acquisition was standardized within our institution, the use of a blood glucose threshold of <200 mg/dL, while consistent with routine clinical practice guidelines, may have influenced the FDG uptake measurements and introduced the possibility of underestimating the metabolic activity in selected patients. Additionally, unmeasured confounders affecting serum albumin levels could not be fully excluded. Serum albumin levels may be influenced by multiple non-oncologic factors, including nutritional status, systemic inflammation, hepatic dysfunction, renal disease, and other chronic comorbid conditions. Due to the retrospective nature of the study, comprehensive adjustment for all such factors was not feasible, and this may have affected the accuracy of albumin-based risk stratification. Given the limited number of progression events relative to the number of evaluated covariates, the multivariable estimates should be interpreted cautiously, particularly for variables demonstrating large effect sizes. Margin status and detailed tumor regression grading were not uniformly available in the retrospective records and therefore could not be reliably incorporated into the comparative or multivariable analyses. We selected PFS as the primary endpoint because it represents an earlier and clinically meaningful indicator of disease behavior following curative-intent therapy, whereas overall survival may be influenced by subsequent treatment lines. Prospective multicenter studies with harmonized imaging protocols are warranted to confirm the reproducibility and generalizability of these findings. Although additional time-dependent ROC analyses were performed, the ROC-derived cutoff value was not externally validated or internally corrected using bootstrap resampling, and therefore should still be interpreted as exploratory. In addition, the comparative modeling analyses performed in this study require further evaluation in larger prospective cohorts.

## 5. Conclusions

The pre-treatment PET SUVmax/albumin ratio was significantly associated with PFS in patients with gastric cancer treated with neoadjuvant FLOT followed by curative gastrectomy. These findings suggest that the PET SUVmax/albumin ratio may represent an exploratory composite prognostic biomarker integrating tumor-related metabolic information and host-related nutritional/inflammatory status. Further prospective multicenter studies are required to externally validate these findings.

## Figures and Tables

**Table 1:** Baseline clinicopathological and metabolic characteristics according to recurrence status.

Variable		Overall (*n* = 104)	No Recurrence (*n* = 58)	Recurrence (*n* = 46)	*p*-Value
**Age, years (mean ± SD)**		61.18 ± 9.98	62.48 ± 10.08	59.54 ± 9.72	0.137
**Sex, *n* (%)**	Male	71 (68.3)	40 (69.0)	31 (67.4)	0.864
	Female	33 (31.7)	18 (31.0)	15 (32.6)	
**ECOG, *n* (%)**	0–1	83 (79.8)	47 (81.0)	36 (78.3)	0.219
	≥2	21 (20.2)	11 (19.0)	10 (21.7)	
**BMI, kg/m^2^ (mean ± SD)**		23.1 ± 2.7	24.12 ± 2.19	21.87 ± 2.66	**<0.001**
**Tumor localization, *n* (%)**	Antrum	48 (46.2)	25 (43.1)	23 (50.0)	**0.007**
	Corpus	13 (12.5)	7 (12.1)	6 (13.0)	
	Cardia	22 (21.2)	18 (31.0)	4 (8.7)	
	Pylorus	9 (8.7)	6 (10.3)	3 (6.5)	
	Diffuse	12 (11.5)	2 (3.4)	10 (21.7)	
**Histological subtype, *n* (%)**	Adenocarcinoma	71 (68.3)	43 (74.1)	28 (60.9)	0.342
	Mucinous	6 (5.8)	3 (5.2)	3 (6.5)	
	Signet-ring cell	27 (26.0)	12 (20.7)	15 (32.6)	
**Tumor differentiation, *n* (%)**	Well	4 (3.8)	3 (5.2)	1 (2.2)	0.057
	Moderate	32 (30.8)	23 (39.7)	9 (19.6)	
	Poor	53 (51.0)	27 (46.6)	26 (56.5)	
	Undifferentiated	15 (14.4)	5 (8.6)	10 (21.7)	
**LVI, *n* (%)**	Negative	48 (46.2)	35 (60.3)	13 (28.3)	**0.001**
	Positive	56 (53.8)	23 (39.7)	33 (71.7)	
**PNI, *n* (%)**	Negative	48 (46.2)	33 (56.9)	15 (32.6)	**0.014**
	Positive	56 (53.8)	25 (43.1)	31 (67.4)	
**Type of surgical resection, *n* (%)**	Total	73 (70.2)	35 (60.3)	38 (82.6)	**0.014**
	Subtotal	31 (29.8)	23 (39.7)	8 (17.4)	
**Extent of lymphadenectomy, *n* (%)**	D1	2 (1.9)	2 (3.5)	0 (0.0)	0.502
	D2	102 (98.1)	56 (96.5)	46 (100)	
**ypT stage, *n* (%)**	0	12 (11.5)	10 (17.2)	2 (4.3)	**0.014**
	1	12 (11.5)	10 (17.2)	2 (4.3)	
	2	14 (13.5)	6 (10.3)	8 (17.4)	
	3	43 (41.3)	24 (41.4)	19 (41.3)	
	4	23 (22.1)	8 (13.8)	15 (32.6)	
**ypN stage, *n* (%)**	ypN0	25 (24.0)	23 (39.7)	2 (4.3)	**<0.001**
	ypN1	22 (21.2)	12 (20.7)	10 (21.7)	
	ypN2	27 (26.0)	14 (24.1)	13 (28.3)	
	ypN3	30 (28.8)	9 (15.5)	21 (45.7)	
**Pathological complete response (pCR), *n* (%)**	No	94 (90.4)	50 (86.2)	44 (95.7)	0.179
	Yes	10 (9.6)	8 (13.8)	2 (4.3)	
**Retrieved lymph nodes, median (IQR)**		20.5 (16–30.75)	18.5 (14.75–26)	24 (18–32)	**0.018**
**Pathologically positive lymph nodes, median (IQR)**		3 (1–7.75)	1 (0–4.25)	5 (2–9.25)	**<0.001**
**Adjuvant FLOT cycles, median (IQR)**		4 (4–4)	4 (4–4)	4 (3–4)	**0.001**
**HER2 status (FISH), *n* (%)**	Negative	98 (94.2)	54 (93.1)	44 (95.7)	0.691
	Positive	6 (5.8)	4 (6.9)	2 (4.3)	
**MSI status, *n* (%)**	MSS	94 (90.4)	50 (86.2)	44 (95.7)	0.227
	MSI-H	9 (8.7)	7 (12.1)	2 (4.3)	
**CRP (median [IQR])**		2.20 [0.90–7.90]	2.05 [1.03–5.90]	2.24 [0.81–8.75]	0.797
**PET SUVmax (median [IQR])**		8.20 [5.8–10.8]	7.25 [5.1–9.28]	10.05 [6.9–12.4]	**0.001**
**Serum albumin, g/dL (median [IQR])**		3.80 [3.4–4.0]	4.00 [3.70–4.13]	3.50 [3.18–3.80]	**<0.001**
**PET SUVmax/albumin ratio (median [IQR])**		2.18 [1.48–3.05]	1.85 [1.34–2.48]	2.97 [1.96–3.83]	**<0.001**

ECOG: Eastern Cooperative Oncology Group; BMI: body mass index; LVI: lymphovascular invasion; PNI: perineural invasion; IQR: Interquartile range; MSI: microsatellite instability; MSS: microsatellite stable; CRP: C-reactive protein; SUVmax: maximum standardized uptake value.

**Table 2:** Univariate and multivariate Cox regression analyses for progression-free survival.

Variable	Univariate Cox HR (95% CI)	*p*-Value	Multivariate Cox HR (95% CI)	*p*-Value
**LVI positive**	2.87 (1.50–5.48)	**0.001**	1.76 (0.73–4.23)	0.206
**PNI positive**	1.97 (1.06–3.66)	**0.032**		
**Tumor localization**		**0.024**		**0.017**
**Corpus vs. Antrum**	0.89 (0.36–2.18)	0.792	0.27 (0.09–0.85)	**0.026**
**Cardia vs. Antrum**	0.30 (0.10–0.87)	**0.027**	0.15 (0.05–0.52)	**0.002**
**Pylorus vs. Antrum**	0.61 (0.18–2.03)	0.415	0.54 (0.13–2.14)	0.377
**Diffuse vs. Antrum**	1.99 (0.95–4.21)	0.070	0.84 (0.34–2.07)	0.703
**Type of surgical resection (Subtotal vs. Total)**	0.38 (0.18–0.82)	**0.014**	0.17 (0.06–0.43)	**<0.001**
**PET SUVmax**	1.17 (1.09–1.25)	**<0.001**		
**PET SUVmax/Albumin**	1.45 (1.25–1.69)	**<0.001**	1.71 (1.33–2.21)	**<0.001**

HR: hazard ratio; CI, confidence interval; LVI: lymphovascular invasion; PNI: perineural invasion; SUVmax: maximum standardized uptake value.

**Table 3:** Exploratory comparative multivariable Cox regression models for progression-free survival.

Model	Variable	HR (95% CI)	*p*-Value
**Model 1**	PET SUVmax	1.22 (1.12–1.33)	<0.001
**Model 2**	Serum albumin	0.23 (0.12–0.44)	<0.001
**Model 3**	PET SUVmax	1.15 (1.04–1.26)	0.004
	Serum albumin	0.37 (0.19–0.75)	0.006
**Model 4**	PET SUVmax/Albumin ratio	1.66 (1.37–2.01)	<0.001

## Data Availability

The data presented in this study are available upon request from the corresponding author. The data are not publicly available due to ethical and privacy restrictions.
